# Partially Obstructed Urethral Strictures Due to Balanitis Xerotica Obliterans Improved by the Use of Topical Tacrolimus: Experience at a Tertiary Care Centre

**DOI:** 10.7759/cureus.76678

**Published:** 2024-12-31

**Authors:** Ahsan Ahmad, Md Zaid Imbisat, Qurana Khatoon

**Affiliations:** 1 Urology, Indira Gandhi Institute of Medical Sciences, Patna, IND; 2 Surgery/Urology, Madhubani Medical College and Hospital, Madhubani, IND; 3 Pathology, Madhubani Medical College and Hospital, Madhubani, IND

**Keywords:** balanitis xerotica obliterans, immunomodulator, tacrolimus, topical, topical tacrolimus 0.1%, urethral stricture

## Abstract

Introduction

Balanitis xerotica obliterans (BXO) can cause phimosis, meatal stenosis, and urethral strictures. However, management of these conditions in BXO patients is difficult. Surgical interventions, with their own risks and complications, demonstrate higher rates of disease recurrence. Recently, topical applications of steroids and immunomodulators have been evaluated for their role in the management of urethral strictures associated with BXO. In this study, we evaluated the role of topical application of tacrolimus in urethral strictures associated with BXO.

Materials and methods

This was a prospective study on male patients having urethral strictures associated with BXO. Patients were thoroughly evaluated and advised topical application of 0.1% tacrolimus. They were then reevaluated at six weeks. If the effects of tacrolimus were found to be satisfactory, then patients were advised to continue this treatment for three months. After three months, the patients were again evaluated. At the time of each reevaluation, changes in uroflowmetry and International Prostate Symptom Score (IPSS) were noted.

Results

A total of 53 patients were included in this study. The mean values of pre- and post-tacrolimus maximum urinary flow rate on uroflowmetry(Q_max_) were 12.00±1.43 m/s and 15.26±3.14 m/s, respectively (p<0.001). The mean values of pre- and post-tacrolimus IPSS scores were 18.55±2.28 and 13.04±4.72, respectively (p<0.001). Based on the results, the application of tacrolimus was found to be ineffective in those with strictures >2 cm in length. Of the 53 patients included in the study, 21 required surgical intervention, as their response to tacrolimus was unsatisfactory.

Conclusion

Topical application of tacrolimus is a safe and feasible treatment option for short-segment (≤2cm) urethral strictures associated with BXO.

## Introduction

Balanitis xerotica obliterans (BXO), a male genital variant of lichen sclerosus et atrophicus (LSA), was first described in 1928 by Stühmer [1]. Although its exact etiology is unknown, growing evidence suggests autoimmune influence, genetic predisposition, inflammatory insults, various infections, and trauma [2,3]. Balanitis xerotica obliterans is most commonly found in patients aged 40-60 years of age [4].

Areas affected by BXO usually involve the foreskin and penile glans. Common physical examination findings include erythematous changes or white hypopigmented lesions. Most patients present with phimosis with varying degrees of difficulty or inability to retract the foreskin [5]. Meatal stenosis and subsequent urethral stricture disease are common outcomes of this condition [6-8].

Differential diagnosis includes neoplastic processes, autoimmune diseases, contact dermatitis, psoriasis, Zoon balanitis, leukoplakia, and fixed drug reactions [9]. Histopathologically, the prevalent lesions found in BXO are epithelial hyperplasia, atrophy, penile intraepithelial neoplasia, basal cell vacuolization, lamina propria sclerosis, and variable patterns of lymphocytic infiltration [10].

Management of BXO remains challenging. When only genital skin is involved, topical use of steroids and immunomodulators has shown varying success rates. However, treatment becomes more challenging when the urethra is also involved. Surgical options include meatoplasty, urethral dilatation, and urethroplasties, but urethral strictures associated with BXO have a higher recurrence rate after surgical interventions [11-13]. 

Recently, topical application of different immunomodulators and steroids has been evaluated for its role in urethral stricture disease associated with BXO [6-8]. Tacrolimus is an immunomodulator that prevents the production of interleukin-2 (IL-2) and consequent T-cell activation, which controls the inflammatory process and is used in many inflammatory skin diseases [14]. The aim of this study is to evaluate the role of topical application of tacrolimus in urethral strictures associated with BXO.

## Materials and methods

This was a prospective study done in the Department of Urology of Indira Gandhi Institute of Medical Sciences, a tertiary care center in Patna, eastern India, between April 2022 and June 2024. Male patients who were >18 years of age, presented with lower urinary tract symptoms due to urethral stricture and biopsy-proven BXO, and provided consent to participate in the study were included. Patients with complete urethral obstruction, recurrent urethral stricture, and known cases of benign prostatic hyperplasia, neurogenic bladder, any malignancy, obstructive uropathy, raised serum creatinine, urinary bladder stones, and immunocompromised conditions were excluded.

The study was approved by the Institutional Ethics Committee of Indira Gandhi Institute of Medical Sciences (letter number: 423/IEC/IGIMS/2022).

For each patient, a detailed history was taken, a physical examination was done, and an International Prostate Symptom Score (IPSS) was given. Uroflowmetry, ultrasonography (kidney, ureter, and bladder), serum creatinine, retrograde urethrogram (RGU), and micturating cystourethrogram (MCU) were done in all cases. All patients included in the study were given a local application of 0.1% tacrolimus twice daily for six weeks. This local application of tacrolimus included application over the prepuce and glans penis as well as intra-urethral instillation for its application on urethral mucosa. At six weeks, patients were re-evaluated. Symptomatic improvement and adverse effects were asked about, and a local examination was done. The IPSS scoring, uroflowmetry, ultrasonography, and RGU-MCU were repeated. Any change in IPSS or maximum urinary flow rate (Q_max_) was noted, as well as changes in uroflowmetry pattern and improvement in the skin color of the glans and prepuce. In case of improvement, patients were continued on tacrolimus once daily for three months, after which patients were again evaluated for further improvement and kept in follow-up. All the patients had a follow-up of at least six months.

Statistical analysis was done using IBM SPSS version 20.0 (IBM Corp., Armonk, NY). The mean and standard deviation of numerical variables were calculated. A paired t-test was used to compare the effect of tacrolimus on IPSS and Q_max_. P-value < 0.05 was considered statistically significant. 

## Results

During the study period (with a mandatory follow-up of six months), a total of 53 patients were included. The mean (±standard deviation) age of the patients was 40.43±5.43 years. Most of the patients were in the age range of 41-50 years. On RGU, 40 (75.5%) patients had stricture <2 cm in length, and 13 (24.5%) patients had stricture ≥2 cm in length. The mean pre-intervention Q_max_ was 12.00±1.43 m/s, and the mean post-intervention Q_max_ was 15.26±3.14 m/s (p<0.001), with this difference being statistically significant. The mean pre-intervention IPSS was 18.55±2.28, and the mean post-intervention IPSS was 13.04±4.72 (p<0.001), with this difference being statistically significant, as seen in Table 1.

**Table 1:** Effect of tacrolimus on all 53 patients included in the study Q_max_: maximum urinary flow rate; IPSS: International Prostate Symptom Score *A paired t-test was applied to obtain p-values and p<0.05 was considered statistically significant.

	Pre-tacrolimus		Post tacrolimus		p-value*
	Mean value	Standard deviation	Mean value	Standard deviation	
Q_max_ (m/s)	12.00	1.43	15.26	3.14	<0.001
IPSS	18.55	2.28	13.04	4.72	<0.001

Out of our 53 patients, 21 (39.6%) needed surgical intervention (meatoplasty, single-stage buccal mucosal graft urethroplasty, and perineal urethrostomy), as their response to tacrolimus was not satisfactory. All 13 (24.5%) patients with stricture ≥2 cm in length needed surgical intervention. Out of 40 (75.5%) patients having strictures <2 cm in length, seven had no satisfactory response to tacrolimus and were managed by surgical intervention. One patient with a stricture <2 cm in length needed surgical intervention in the follow-up period due to the recurrence of symptoms and stricture.

The mean pre-tacrolimus Q_max_ in patients with <2 cm stricture was 12.60±1.01 m/s, and the mean post-tacrolimus Q_max_ was 16.82±1.43 m/s (p < 0.001), whereas, for patients with ≥2 cm stricture, the mean pre-tacrolimus Q_max_ was 10.15±0.80 m/s, and the mean post-tacrolimus Q_max_ was 10.46±1.71 (p = 0.472), with these differences being statistically insignificant, as seen in Tables 2-3.

**Table 2:** Effect of tacrolimus on strictures <2 cm in length (40 patients) Q_max_: maximum urinary flow rate; IPSS: International Prostate Symptom Score *A paired t-test was applied to obtain p-values and p<0.05 was considered statistically significant.

	Pre-tacrolimus		Post tacrolimus		p-value*
	Mean value	Standard deviation	Mean value	Standard deviation	
Q_max_ (m/s)	12.60	1.01	16.82	1.43	<0.001
IPSS	17.85	2.06	10.87	2.86	<0.001

**Table 3:** Effect of tacrolimus on strictures ≥2 cm in length (13 patients) Q_max_: maximum urinary flow rate; IPSS: International Prostate Symptom Score *A paired t-test was applied to obtain p values and p<0.05 was considered statistically significant.

	Pre-tacrolimus		Post tacrolimus		p-value*
	Mean value	Standard deviation	Mean value	Standard deviation	
Q_max_ (m/s)	10.15	0.80	10.46	1.71	0.472
IPSS	20.69	1.44	19.69	2.56	0.115

The mean pre-tacrolimus IPSS in patients with strictures <2 cm was 17.85±2.06, and the mean post-tacrolimus IPSS was 10.87±2.86 (p < 0.001), whereas for patients with ≥ 2 cm stricture, the mean pre-tacrolimus IPSS was 20.69±1.44, and the mean post-tacrolimus IPSS was 19.69±2.56 (p = 0.115), with these differences being statistically insignificant.

None of our patients reported any major adverse effects. Some patients reported mild irritation of the prepuce and glans skin. Mild skin erythema was noted in a few patients.

## Discussion

Management of urethral strictures associated with BXO is still a challenge, with the progressive nature of the disease and its high rate of recurrence further complicating treatment. Depending on the stricture length and location urethral dilatation, meatoplasty, internal urethrotomy, one-stage or two-stage urethroplasty, and perineal urethrostomy are surgical options [15,16].

Kulkarni et al. have reported encouraging results for urethroplasty in long-segment strictures associated with BXO, but urethroplasty is associated with significant adverse events, including erectile dysfunction, donor site complications, urethral diverticulum, and chordee. Moreover, urethroplasty is a major surgery requiring patient fitness for the intervention [16,17]. Therefore, conservative management of urethral strictures associated with BXO is highly desired.

Pharmacological treatment has been proven to be highly effective in the treatment of BXO involving genital skin [15]. Hengge et al., in their multicentric study done in 2006, reported topical tacrolimus ointment to be an effective and safe treatment for long-standing active lichen sclerosus. In their study, clearance of active lichen sclerosus was reported in 43% of patients at 24 weeks of treatment, and partial response was seen in 34% of the patients [18].

Topical corticosteroids (e.g., clobetasol propionate ointment) are a known and effective treatment for BXO involving the glans and prepuce [19]. For example,

Kim et al. in their study reported the safety and efficacy of topical tacrolimus (a calcineurin inhibitor) in the management of genital lichen sclerosus [20]. Tausch et al. also reported good results when using topical clobetasol in BXO involving the skin and meatus [21].

In addition to the encouraging pharmacotherapy results of topical corticosteroids on genital skins involved with BXO, their use has also been evaluated for urethral strictures associated with BXO or genital lichen sclerosus. In 2008, Ebert et al. did a pilot study to determine the safety and efficacy of tacrolimus 0.1% ointment in the postoperative period in proven cases of LSA. Topical application of tacrolimus 0.1% was found to be safe and effective in disease control during the postoperative period [22].

Results of a 2010 study by Karatas et al. strongly indicated that tacrolimus-eluting stents may be useful for the management of recurrent urethral stricture [23]. In 2011, Mallick et al. studied patients attending the outpatient department with typical clinical features of BXO. Their study demonstrated symptomatic relief in 53.33% of cases treated with tacrolimus ointment [24].

Dey et al. used intraurethral instillations of tacrolimus 0.03% in 20 patients with urethral stricture and biopsy-proven BXO, with 75% of the patients responding favorably to the treatment; four of their patients did not respond well, and one patient required urethroplasty due to recurrent urinary tract infections [25].

Choudhury et al. reported that both clobetasol and tacrolimus were effective in the improvement of symptom score, Q_max_, and local external appearance. Although post-intervention intergroup differences in IPSS were not significant, post-intervention intergroup differences in Q_max_ were statistically significant in favor of clobetasol (p = 0.007) [26].

Our study evaluated the safety and efficacy of local applications of tacrolimus 0.1% in the management of urethral strictures associated with BXO. The use of tacrolimus significantly improved Q_max_ and IPSS, which has also been previously reported in the literature [24-26].

We also evaluated the effect of tacrolimus and its dependence on stricture length. Application of tacrolimus was not effective in patients with strictures ≥2 cm in length, and these patients required surgical interventions. Of the 40 patients with strictures <2 cm in length, only eight (seven patients due to no response and one patient due to recurrence in follow-up) required surgical intervention.

Study limitations

This study is a single-group study. It had a mandatory follow-up of only six months. Additional comparative studies with long-term follow-up are needed to fully understand the risk of recurrence after any initially satisfactory improvement.

## Conclusions

Tacrolimus, being an immunomodulator, could be an effective conservative treatment option for short-segment (≤2 cm) urethral strictures due to BXO. Its topical application is feasible and safe, and its use avoids the complications of surgical interventions. However, further studies with longer-term follow-up are necessary. These future studies may further establish the possible effective and satisfactory role of tacrolimus in these conditions.

## References

[1] Charlton OA, Smith SD (2019). Balanitis xerotica obliterans: a review of diagnosis and management. Int J Dermatol.

[2] Celis S, Reed F, Murphy F, Adams S, Gillick J, Abdelhafeez AH, Lopez PJ (2014). Balanitis xerotica obliterans in children and adolescents: a literature review and clinical series. J Pediatr Urol.

[3] Boksh K, Patwardhan N (2017). Balanitis xerotica obliterans: has its diagnostic accuracy improved with time?. JRSM Open.

[4] Fekete GL, Schwarzkopf-Kolb D, Brihan I, Boda D, Fekete L (2022). Balanitis xerotica obliterans: an observational, descriptive and retrospective clinical study. Exp Ther Med.

[5] Thakur RS, Pinjala P, Babu M (2015). Balanitis xerotica obliterans Bxo-mimicking vitiligo. IOSR J Dent Med Sci.

[6] Nguyen AT, Holland AJ (2020). Balanitis xerotica obliterans: an update for clinicians. Eur J Pediatr.

[7] Gargollo PC (2013). Phimosis, meatal stenosis, and BXO. Pediatric Urology.

[8] Clouston D, Hall A, Lawrentschuk N (2011). Penile lichen sclerosus (balanitis xerotica obliterans). BJU Int.

[9] Fergus KB, Lee AW, Baradaran N (2020). Pathophysiology, clinical manifestations, and treatment of lichen sclerosus: a systematic review. Urology.

[10] Piris A, Sanchez DF, Fernandez-Nestosa MJ (2020). Topographical evaluation of penile lichen sclerosus reveals a lymphocytic depleted variant, preferentially associated with neoplasia: a report of 200 cases. Int J Surg Pathol.

[11] Prakash S, Mahesh M, Kumar V, Sarwar MF, Hasan A (2024). Effect of topical tacrolimus in patients of non obliterative anterior urethral stricture due to BXO. Res J Med Sci.

[12] Kurtzman JT, Blum R, Brandes SB (2021). One-stage buccal mucosal graft urethroplasty for lichen sclerosus-related urethral stricture disease: a systematic review and pooled proportional meta-analysis. J Urol.

[13] Chen L, Hou R, Feng C, Zhang X, Li D, Chen J, Hu B (2017). Establishment of the U.L.T.R.A. measurement rating system for anterior urethral stricture. Int Urol Nephrol.

[14] Assmann T, Ruzicka T (2002). New immunosuppressive drugs in dermatology (mycophenolate mofetil, tacrolimus): unapproved uses, dosages, or indications. Clin Dermatol.

[15] Chung AS, Suarez OA (2020). Current treatment of lichen sclerosus and stricture. World J Urol.

[16] Kulkarni S, Barbagli G, Kirpekar D, Mirri F, Lazzeri M (2009). Lichen sclerosus of the male genitalia and urethra: surgical options and results in a multicenter international experience with 215 patients. Eur Urol.

[17] Al-Qudah HS, Santucci RA (2005). Extended complications of urethroplasty. Int Braz J Urol.

[18] Hengge UR, Krause W, Hofmann H (2006). Multicentre, phase II trial on the safety and efficacy of topical tacrolimus ointment for the treatment of lichen sclerosus. Br J Dermatol.

[19] Kwok R, Shah TT, Minhas S (2020). Recent advances in understanding and managing lichen sclerosus. F1000Res.

[20] Kim GW, Park HJ, Kim HS, Kim SH, Ko HC, Kim BS, Kim MB (2012). Topical tacrolimus ointment for the treatment of lichen sclerosus, comparing genital and extragenital involvement. J Dermatol.

[21] Tausch TJ, Peterson AC (2012). Early aggressive treatment of lichen sclerosus may prevent disease progression. J Urol.

[22] Ebert AK, Rösch WH, Vogt T (2008). Safety and tolerability of adjuvant topical tacrolimus treatment in boys with lichen sclerosus: a prospective phase 2 study. Eur Urol.

[23] Karatas OF, Cimentepe E, Bayrak O, Unal D (2010). A new application for urethral strictures: tacrolimus-eluting stent. J Endourol.

[24] Mallick A, Majhi T, Basu S, Pal DK (2013). Balanitis xerotica obliterans, the topical application of tacrolimus ointment, and the result: an institutional study. UroToday Int J.

[25] Dey RK, Khan I, Khan D (2017). Study of intraurethral instillation of tacrolimus for urethral involvement following lichen sclerosus. Int J Sci Study.

[26] Choudhury S, Khare E, Pal DK (2023). Comparative effect of intraurethral clobetasol and tacrolimus in lichen sclerosus-associated urethral stricture disease. Urol Ann.

